# Effect of a Female External Urinary Catheter on Incidence of Catheter-Associated Urinary Tract Infection

**DOI:** 10.7759/cureus.11113

**Published:** 2020-10-23

**Authors:** Jillian Zavodnick, Caitlin Harley, Kelly Zabriskie, Yasmin Brahmbhatt

**Affiliations:** 1 Department of Internal Medicine, Thomas Jefferson University, Philadelphia, USA; 2 Department of Nursing, Thomas Jefferson University, Philadelphia, USA; 3 Department of Infection Control, Thomas Jefferson University, Philadelphia, USA

**Keywords:** cauti, hospital acquired infection, external urinary catheter, female external urinary catheter, cauti prevention, infection control, nosocomial infection

## Abstract

Background

Catheter-associated urinary tract infections (CAUTIs) can be fatal, and are a source of avoidable expense for patients and hospitals. Prolonged catheterization increases infection risk, and avoiding catheters is crucial for infection prevention. Male external urinary catheters are recommended as a tool to prevent the need for indwelling catheterization. Female external urinary catheters (FEUCs) have intermittently been marketed without wide adoption; one has recently become available but published data is limited.

Objective

This retrospective observational study was conducted to investigate the effect of FEUCs on indwelling catheter use and female CAUTIs.

Methods

FEUCs were introduced to intensive care units. CAUTI rates and indwelling catheter days were obtained before and after the introduction of the devices.

Results

CAUTI rates decreased from 3.14 per 1000 catheter days to 1.42 per 1000 catheter days (p=0.013). Female indwelling catheter days decreased, while overall intensive care patient days increased.

Conclusions

Introduction of a FEUC was associated with a statistically significant decrease in CAUTI rate among female intensive care patients. The FEUC may prevent the need for indwelling catheters in some situations.

## Introduction

Indwelling urinary catheters (IUC) are commonly used in hospitals, and have a number of potential complications including catheter-associated urinary tract infections (CAUTIs) [1]. CAUTIs increase mortality, length of stay, and costs, with the cost of a single CAUTI recently estimated to be over $10,000 for a patient in intensive care; prevention is therefore a major focus for hospitals [2-4]. Longer duration of indwelling catheterization increases CAUTI risk, and avoidance of IUCs when feasible is an important part of CAUTI prevention [5]. Educating hospital staff on the benefits of IUC avoidance and removal [6], as well as restricting IUC use and increasing access to alternatives to catheterization, have been well-studied methods for decreasing CAUTIs [7,8]. Male external urinary catheters have long been available, and are recommended by multiple society guidelines and as part of a published preventive bundle to decrease CAUTIs [5,9-11]. Female external urinary catheters (FEUCs) have intermittently been marketed but never widely adopted [9,12-15]. The PureWick FEUC (Bard and Purewick, Becton, Dickinson and Company, Franklin Lakes, New Jersey) has recently become available, but published data is so far limited to a single case series without systematic outcomes, a limited local investigation showing a decrease in IUC utilization after implementation without a decrease in CAUTI, and one inpatient study demonstrating an initial decrease in CAUTI which was not sustained after one year [9,16-18].

CAUTI rates at our institution decreased over the past ten years with the introduction of new prevention measures (e.g. nurse-driven catheter removal protocol), but then reached a plateau once these measures had been consistently adopted. We began performing root cause analysis on all CAUTIs to determine factors leading to the infection, with a goal of targeting future interventions to further decrease CAUTIs. Among other findings, we recently learned that the majority of IUCs were placed for the purpose of close monitoring of urine output. We hypothesized that the introduction of an FEUC would decrease utilization of IUCs, and that this would prevent CAUTIs.

As our outcome measure, we compared CAUTI rates in female ICU patients before and after the product became available. We measured catheter days in female ICU patients as a process measure, hypothesizing that FEUC availability would decrease IUC use as the mechanism of decreasing CAUTI.

## Materials and methods

Setting

Thomas Jefferson University Hospital (TJUH) is a large urban academic medical center closely associated with Jefferson Hospital for Neuroscience (JHN); these hospitals report jointly for some measures. Together these hospitals contain nine intensive care units. The hospitals have long had a nurse-driven IUC protocol enabling removal without a physician order when patients no longer meet criteria for IUC use, a practice shown to decrease CAUTI [19]. Jefferson’s standardized infection ratio, a measure of CAUTI prevalence, was 0.59 in the year prior to this study.

Study design

We performed a retrospective observational study. Participants included all adult ICU patients from January 1, 2017 to December 31, 2019, a total of 89,856 patient days. As our main outcome, we compared CAUTI rates in female ICU patients in the year before (Jan 1, 2017 to December 31, 2017) and the two years after (Jan 1, 2018 to December 31, 2019) the FEUC became available at our hospital using the National Healthcare Safety Network (NSHN) measure of CAUTI per 1,000 catheter days [20]. When calculating CAUTI rates, IUC days, and hospital-acquired pressure injury (HAPI) rates, male patients were excluded.

The hypothesized mechanism for decreased CAUTI after FEUC introduction was decreased IUC use. To confirm this, we examined IUC days in female patients, calculated as a count of the number of female patients with IUC presence charted by the nurse each day of the study period. A decrease in IUC days with the introduction of the FEUC would suggest the avoidance of IUC use, while no decrease would suggest that the FEUC was being adopted primarily in patients who would not have had an IUC.

HAPI was analyzed in the same population over the same time frame as a secondary outcome and balancing measure. The measure was defined as a pressure-induced mucosal injury, non-mucosal ulcer of stage one through four, unstageable ulcer, or deep tissue injury [21]. If the pressure injury was related to a medical device, this was recorded as well. The adoption of the FEUC for patients with moisture dermatitis or at risk of skin breakdown was not anticipated to decrease CAUTI rates as these patients would not otherwise have had IUCs, but might be expected to decrease HAPIs, making HAPI a useful secondary outcome. However, since our HAPI data includes device-related pressure injuries, an increase in device-related HAPI with the introduction of an FEUC could be evidence of unintended consequences of device adoption. We did not measure moisture dermatitis rates directly.

We were not able to directly measure FEUC days or how many patients had an FEUC placed. Information on ICU patient days was collected to control for the impact of patient volume on IUC days; information on female-only ICU days was not available, so male and female patients are included in this measure.

CAUTI data was obtained by infection control personnel from an Epic data report of positive urine cultures, which infection control staff used to determine whether CAUTI criteria were met. It was then analyzed using the NHSN data reporting tool and Microsoft Excel. HAPI data was obtained by staff nurses trained as “skin champions” during a monthly assessment of all ICU patients, and was analyzed using Microsoft Excel and SPSS. The two-sided Fisher’s exact test was used to determine statistical significance.

Intervention

**Figure 1:**
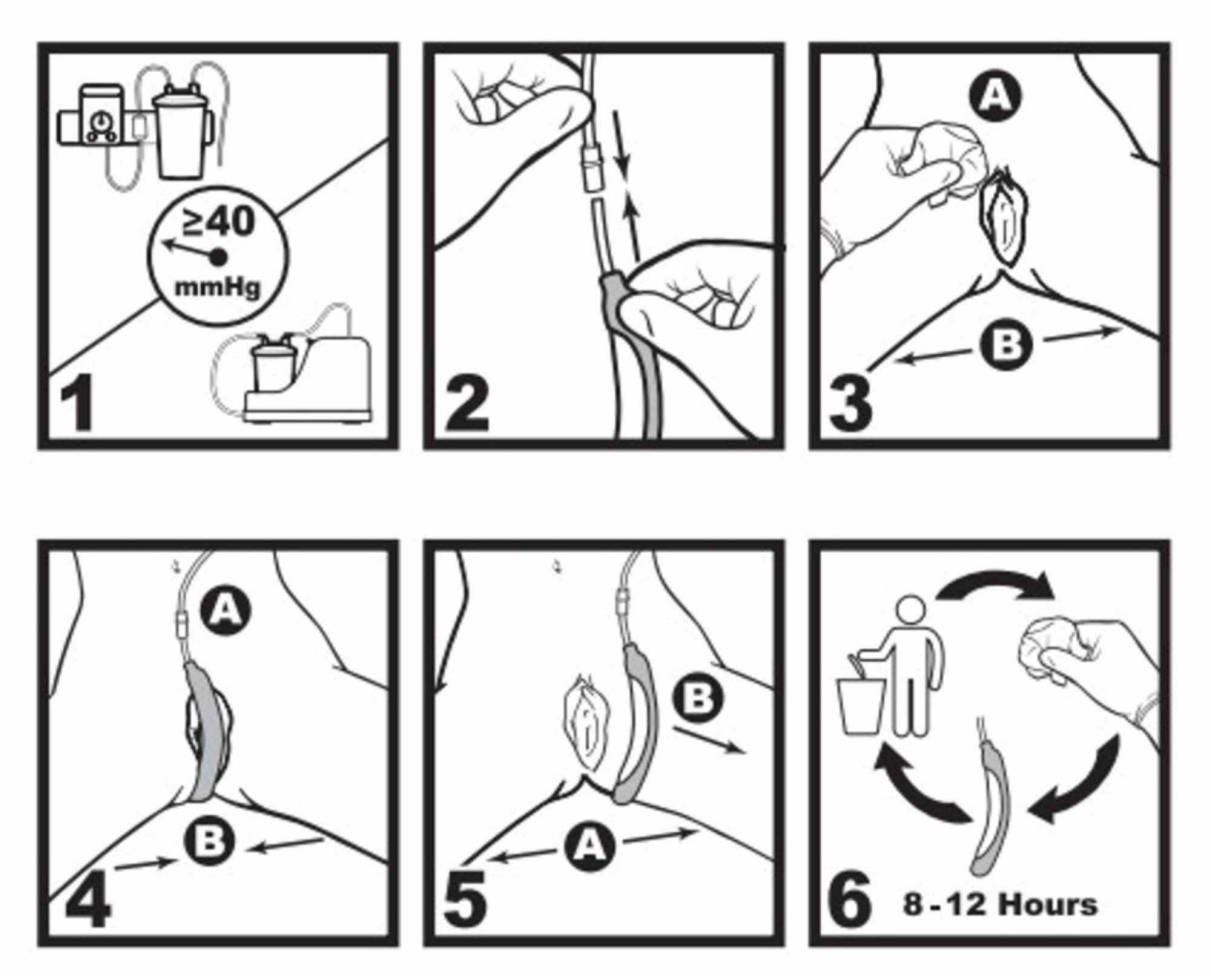
Schematic of the PureWick Urinary Collection System © 2019 BD. Used with permission. Bard and PureWick are trademarks and/or registered trademarks of Becton, Dickson and Company or its affiliates. mmHg = millimeters of mercury

**Figure 2:**
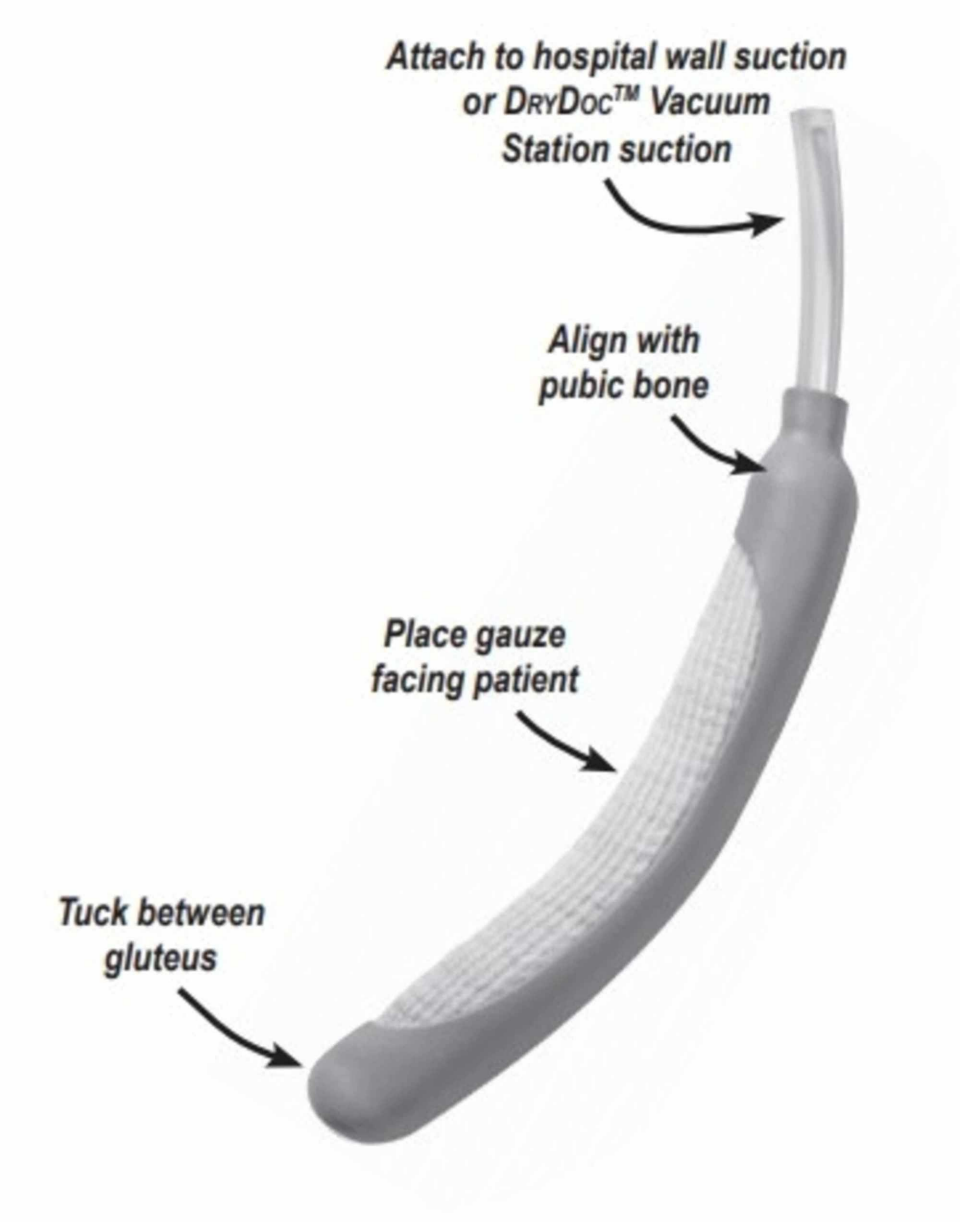
Detailed Image of the Patient Portion of the Female External Urinary Catheter © 2019 BD. Used with permission. Bard and PureWick are trademarks and/or registered trademarks of Becton, Dickson and Company or its affiliates.

The PureWick FEUC was introduced to all ICUs in January of 2018. The product consists of a urine collection segment positioned between the patient’s labia, which is attached to wall suction (Figures 1-2).

Patients cannot ambulate while the FEUC is in place. The decision to use the FEUC in a given patient was nursing-driven, with guidelines given for appropriate indications. The first indication was patients requiring strict monitoring of intake and output without other means of obtaining these measurements; using the FEUC for this indication was anticipated to avoid the need for an indwelling catheter. The second indication was moisture dermatitis or potential for skin breakdown due to incontinence; this is not an indication for an indwelling catheter at our institution, although inappropriate use of catheters for this indication has been described [22]. Using an FEUC for this indication was intended to decrease pressure injuries, and was not expected to decrease IUC use or CAUTI.

The introduction was overseen by the CAUTI Working Group, an interprofessional hospital committee with joint nursing and physician leadership, and representation from infection control professionals. Representatives from the manufacturer trained all ICU nurses in the use of the product. A few nurse members for each unit underwent more in-depth training so that they could serve as “superusers” to continuously educate other staff. Product representatives remained available for questions as nurses began using the FEUC.

The Thomas Jefferson University Institutional Review Board categorized this intervention as work not requiring review.

## Results

In the year prior to FEUC introduction, there were 23 CAUTIs in female ICU patients, a rate of 3.14 per 1000 indwelling catheter days. In the two years after FEUC introduction there were 17 CAUTIs in this population (1.42 events per 1000 indwelling catheter days), p=0.013. A yearly breakdown shows a continued downward trend, with a CAUTI rate of 1.68 in the first post-intervention year, and a rate of 1.17 in the second post-intervention year. There was an 18.2% decrease in ICU female indwelling catheter days, from an average of 610 monthly device days to 499 monthly device days. During this same period, overall ICU patient days increased from a monthly average of 2392 to 2548 (Table 1).

**Table 1:** Female CAUTI Rate and Number of Female IUC Days Before and After Introduction of FEUC CAUTI = catheter associated urinary tract infection. IUC = indwelling urinary catheter. FEUC = female external urinary catheter.

	Before FEUC (January 1, 2017 – December 31, 2017)	After FEUC (January 1, 2018 – November 30, 2019)	P value
Number of CAUTIs	23	17	
Female CAUTI rate (events per 1000 catheter days)	3.14	1.42	0.013
Number of female IUC days	7324	11971	
Number of patient days (male and female)	28702	61154	
Average monthly patient days	2392	2548	
Average monthly female IUC days	610	499	

There were 9 HAPIs in female ICU patients between January 1 and December 31 of 2017 (pre-FEUC) out of 833 patients sampled (prevalence 1.08%). Of these, 4 (44.4%) were related to medical devices. Between January 1, 2018 and November 30, 2019 (post-FEUC; a prevalence assessment was not completed for December of 2019) there were 29 HAPIs in the 1809 female ICU patients sampled (prevalence 1.60%), 11 (37.9%) related to medical devices. Neither the increase in HAPI overall nor the increase in device-associated pressure injuries was statistically significant (p=0.38 and 0.43 respectively) (Table 2).

**Table 2:** Female HAPI and Device-Associated Pressure Injury Prevalence Before and After FEUC HAPI = hospital acquired pressure injury. FEUC = female external urinary catheter.

	Prevalence before FEUC (January 1, 2017 – December 31, 2017)	Prevalence after FEUC (January 1, 2018 – November 30, 2019)	P value
HAPI	1.08% (n=9)	1.60% (n=29)	0.38
HAPI monthly average	0.75	1.26	
Device-associated HAPI	0.49% (n=4)	0.61% (n=11)	0.43

## Discussion

The availability of a FEUC was associated with a statistically significant decrease in CAUTI. After the FEUC became available, IUC days decreased among female ICU patients, even though the number of ICU patient days increased overall. The decrease in IUC days alongside the increase in ICU days suggests that there may be some adoption of the FEUC in place of IUCs for strict intake and output monitoring, a trend which would be expected to decrease CAUTI rates, and has been demonstrated in recently presented data [17].

Data on skin outcomes does not suggest that pressure injuries are being avoided through use of the FEUC. We propose the measurement of skin outcomes during the introduction of this device for two purposes. First, this can serve as a secondary outcome measure for the use of a FEUC in cases of incontinence alone without an indication for an IUC. Second, device-related pressure injuries could be a useful balancing measure, demonstrating unintended consequences of FEUC adoption. However, HAPI is a heterogeneous and multifactorial measure and there are multiple variables besides FEUC adoption which may have led to our increased rates. Many patients at risk for HAPI have no indication for an external or internal urinary catheter, and we expected that any effect of this product on HAPI would be modest at best. HAPI data collection does include device-associated pressure injuries, which are a useful balancing measure for FEUC use; these did not significantly change during the intervention period. Ongoing careful surveillance will be needed to ensure that the FEUC is not associated with increased device-associated pressure injuries.

Informal feedback from patients and nursing has been positive, and prior to the availability of outcomes data, the product had been introduced to all units of the hospital. This was done at the request of non-ICU nurses who were aware of the product, and after feedback from patients who had used the FEUC while in the ICU and were frustrated at the need to discontinue it when transferred to the floors. Even before a statistically significant decrease in CAUTI rates was demonstrated, staff and patient feedback led the hospital to continue making the FEUC available.

There are some limitations to our analysis. We were not able to obtain specific data on FEUC utilization. For the primary outcome of CAUTI, we used the process measure of IUC utilization as a proxy for FEUC use. However, there could be other factors leading to the decrease in IUC use during this time frame. We also noted an increase in ICU days for all patients alongside a trend toward fewer CAUTIs in female ICU patients, the only population expected to be affected by the availability of an FEUC. From this we inferred that the decrease in IUC use was not driven purely by lower patient volumes. This inference depends on two assumptions: first, that female ICU days did not decrease relative to male ICU days over this time period; and second, that there was no difference in the number of patients with a potential IUC indication between the before and after periods. These assumptions are reasonable but cannot be demonstrated by our data.

There are also significant limitations pertaining to skin outcomes. Moisture-associated dermatitis would have been a useful outcome to directly track the effectiveness of the FEUC when used for the indication of skin protection, but was not available. The use of HAPI as a proxy measure ignores patients who might have moisture-associated dermatitis that does not progress to a pressure injury. Data collection for HAPI comes from monthly prevalence surveys and may underestimate device-associated pressure injury, which is an infrequent occurrence. This data is unable to distinguish between types of devices leading to pressure injury; pressure injuries resulting from tracheostomy devices, for example, would be unaffected by our intervention. HAPI assessment was not performed in December of 2019, which is expected to artificially lower the total number of HAPI in the post-intervention period (monthly average and prevalence should remain unaffected). Although we recommend that institutions adopting the FEUC make an effort to measure skin outcomes, our HAPI measurement procedures significantly limit this measure’s use for the purpose of evaluating this device.

An FEUC is a feasible, well-tolerated intervention that is associated with decreased IUC utilization and CAUTI rates. Further study is needed with direct tracking of FEUC utilization by indication, and with measurement of moisture dermatitis. A cost-benefit analysis would be useful in determining the total utility of this product. Further investigation could consider the incorporation of the population CAUTI rate, CAUTI per 10,000 patient days, a measure proposed as an alternative to the NHSN definition in a low-prevalence environment [23].

## Conclusions

The number of IUC days decreased with the availability of the FEUC despite a slight increase in patient days, suggesting that this product may prevent the need for IUC in some situations. There was a statistically significant decrease in CAUTI rate, a meaningful outcome which also further supports the conclusion that IUC use decreased. No change was noted in HAPI, but this analysis was subject to significant limitations. Further quality improvement initiatives that focus on female IUC, HAPI and CAUTI rates with the FEUC product are warranted.
